# Rapid Diagnosis and Treatment of Patients with Acute Type A Aortic Dissection and Malperfusion Syndrome May Normalize Survival to that of Patients with Uncomplicated Type A Aortic Dissection

**DOI:** 10.1055/s-0039-1691790

**Published:** 2019-09-17

**Authors:** Syed Usman Bin Mahmood, Makoto Mori, Jiajun Luo, Yawei Zhang, Basmah Safdar, Andrew Ulrich, Arnar Geirsson, John A. Elefteriades, Abeel A. Mangi

**Affiliations:** 1 Section of Cardiac Surgery, Department of Surgery, Yale University School of Medicine, New Haven, Connecticut; 2 Section of Surgical Outcomes and Epidemiology, Yale School of Public Health, New Haven, Connecticut; 3 Department of Emergency Medicine, Yale University School of Medicine, New Haven, Connecticut

**Keywords:** Type A aortic dissection, malperfusion syndrome, ascending aortic replacement

## Abstract

**Objectives** Malperfusion syndrome in the setting of acute Type A dissection (ATAD) is typically associated with poor prognosis. We evaluated the contemporary outcomes of patients with ATAD presenting with and without malperfusion syndrome who underwent aortic surgery.

**Methods** We performed a single-center, retrospective review of 103 consecutive patients that underwent surgery for ATAD. The cohort was dichotomized by patients with and without malperfusion syndromes. Multivariate and bivariate analyses were performed to evaluate association between the presence of malperfusion syndrome and operative outcomes.

**Results** A total of 29 (28.1%) patients presented with malperfusion syndrome. The 30-day mortality for patients presenting with and without malperfusion was 13.7 and 9.4%, respectively ( *p* = 0.49). Patients with malperfusion syndrome had a shorter mean admission-to-incision interval of 4.3 ± 2.5 hours compared with 6.3 ± 4.6 hours for those without malperfusion ( *p* = 0.02). Difference in 30-day mortality for patients with and without malperfusion syndrome was found to be nonsignificant on multivariate regression analysis (odds ratio: 1.53; 95% confidence interval: 0.40–5.82, *p* = 0.49).

**Conclusions** This series demonstrated that there was nonsignificant difference in early- or midterm outcomes for patients with and without malperfusion syndrome. Patients with malperfusion were taken to the operating room more rapidly than those without, which offers a potential explanation for the comparable outcome of the malperfusion cohort.

## Introduction

Acute Type A aortic dissection (ATAD) is a cataclysmic event requiring emergent surgery. Perioperative mortality for ATAD is inversely proportional to institutional experience, ranging from 16.4 to 27.4%, and averaging around 21.6% in the U.S. 1 2

Malperfusion syndrome significantly compromises the outcomes of ATAD patients and warrants expeditious diagnosis. Malperfusion usually results from extension of the dissection flap into the branch vessel, with static or dynamic narrowing or obstruction of the branch orifice by the flap. 3 The subtle nature of compromised end-organ perfusion and ensuing ischemia may result in diagnostic delays and can result in comparatively higher mortality rates than ATAD without malperfusion. 4 Cases involving renal or mesenteric ischemia are known to have higher (> 50%) postoperative mortality rates. 3 4 5 Surgical mortality for patients presenting with any visceral malperfusion has been recorded to be as high as 43% ± 4%, nearly twice in comparison to the overall ATAD cohort (25% ± 3%). 5

ATAD complicated by malperfusion usually presents with clinical symptoms of acute chest pain, syncope, stroke, limb ischemia, abdominal pain, and/or diarrhea. 6 However, presumably due to the dynamic nature of end-organ malperfusion, some patients present without clear signs of malperfusion, and a high index of suspicion is needed to establish the correct diagnosis in a timely manner, thereby avoiding end-organ infarction. 7 This difficulty in diagnosis and adverse impact on outcome is especially great in regard to intestinal ischemia. Cerebral and extremity malperfusion are usually more obvious.

Most surgical centers perform immediate central aortic replacement surgery for ATAD complicated by malperfusion, reserving peripheral bypass, stent grafting, and fenestration for patients in whom malperfusion remains uncorrected after central repair. The Michigan group approaches intestinal malperfusion first, by endovascular means, delaying central aortic replacement accordingly. Our center applies an acute thoracic emergency protocol for managing cases presenting to the emergency department (ED) with a high index of suspicion for aortic dissection/rupture. This protocol prioritizes triage and diagnosis of such cases to expedite their transfer to the operating room for definitive management.

The importance of emergent surgical intervention for ATAD surgery is well known and forms the basis of management guidelines for these cases. The aim of this article is to characterize contemporary outcomes in ATAD patients with and without malperfusion syndrome who underwent emergent aortic surgeries, with special emphasis on time from diagnosis to treatment.

## Materials and Methods

This study was approved by the Institutional Review Board (HIC#: 2000021950). A single-center, retrospective review of patients undergoing consecutive ascending aortic replacement surgery for ATAD from 2008 to 2017 at the Yale New Haven Hospital (YNHH) was performed. Patients who had complete record of time of admission, time of transfer, and diagnosis were included in the cohort. Initial screening yielded 128 patients with Type A aortic dissection and further exclusions were made for patients with chronic ascending aortic dissection (15 cases), traumatic aortic dissection (7 cases), and incomplete data (3 cases). The final cohort consisted of 103 acute ATAD cases that were surgically managed at YNHH. Time of first presentation to a medical facility and time of incision were recorded for all patients and this interval was defined as the “admission–incision interval.” Diagnosis of malperfusion syndrome was confirmed using the ED physician's diagnostic notes and cardiac surgeon's clinical findings based on examination. For patients who were transferred from outside medical facilities, dissection and malperfusion was confirmed on their respective primary hospital notes or recorded as an observation by the surgeon prior to surgical exploration.

Mood's median test, a nonparametric test, was used to compare time from admission to incision between different groups. Chi-square test was used to examine the difference in categorical variables between patients with and without malperfusion syndrome. Multivariate unconditional logistic regression was employed to evaluate the associations between malperfusion and postoperative outcomes including ventilation over 48 hours, reoperation for bleeding, postoperative stroke, death during hospitalization, and 30-day mortality. Age, race, sex, and body mass index (BMI) were adjusted in all logistic models. The log rank method was used to estimate postoperative overall survival between patients with malperfusion and without malperfusion. All tests were two-sided with a *p* -value less than 0.05 being considered significant. All statistical analyses were performed using SAS 9.4 (SAS Institute Inc., Cary, NC).

## Results

The cohort comprised 71 (68.9%) male and 32 (31.1%) female patients. The overall mean age at the time of surgery was 59.2 ± 14.3 years and BMI was 29.2 ± 6.0 kg/m ^2^ ( Table 1 ). Mean systolic and diastolic blood pressures measured at initial presentation were 131.4 ± 34.6 and 73.9 ± 22.7 mm Hg, respectively; however, 40 patients were on chronic beta-blocker therapy or started on anti-impulse therapy at an outside facility.

Malperfusion syndrome was suspected in 29 (28.2%) patients. Distal lower extremity pulses were not palpable in 48% of these cases. Other patients with malperfusion had clinical indications of cerebral and/or upper extremity ischemia. Abdominal malperfusion was noted as a clinical suspicion in patients with acute abdomen coupled with new-onset diarrhea and abdominal pain.

Overall, 8 (27.5%) patients with malperfusion underwent a “point-of-care” (POC) heart echocardiogram by an ED physician and their mean admission-to-incision interval was 4.8 ± 3.3 hours compared with 4.1 ± 2.3 hours for malperfused patients who did not undergo a POC echo ( *p* = 0.51).

Furthermore, 66 patients also had an intraoperative transesophageal echocardiogram (TEE) performed before initiation of aortic repair. Echocardiogram confirmed presence of moderate-large pericardial effusion in 17 cases with 4 patients in the malperfusion group (13.7%) and 13 (17.5%) in the nonmalperfusion group ( *p* = 0.28). The intraoperative TEE confirmed moderate to severe aortic insufficiency in 24.1% patients with malperfusion syndrome and 25.6% patients without malperfusion ( *p* = 0.87).

**Fig. 1:**
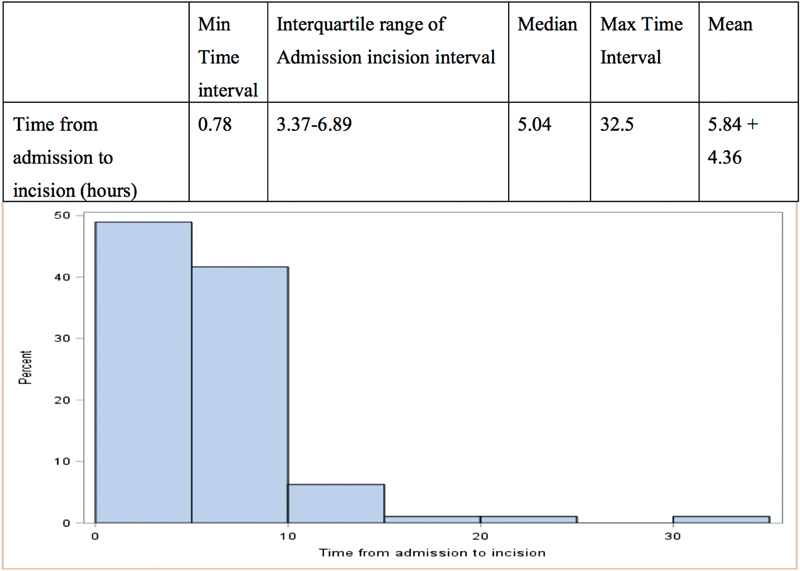
Admission–incision interval for all patients.

Confirmation of diagnosis was made on chest computed tomography (CT) scans with a majority (80.6%) demonstrating Type I DeBakey aortic dissection and the remaining patients Type II dissections. Presence of a dissection flap was confirmed in the ascending aorta of 94 patients (91.2%), aortic arch of 82 patients (79.6%), descending aorta of 61 patients (59.2%), abdominal aorta of 56 patients (54.3%), and iliac arteries of 39 patients (37.8%). The overall mean admission-to-incision interval was 5.8 ± 4.3 hours (median = 5 hours; Fig. 1 ) and the mean duration from admission to CT scan was 1.7 ± 2.5 hours (median = 1 hour).

Patients with malperfusion syndrome had a mean admission-to-incision interval of 4.3 ± 2.5 hours (median: 3.6 hours); those without malperfusion had an average admission-to-incision interval of 6.3 ± 4.6 hours (median: 5.5 hours; *p* = 0.02).

There was no significant difference in the surgical procedure (i.e., central repair and then peripheral revascularization if needed) among ATAD cases with and without malperfusion ( Table 2 ). All patients with malperfusion syndrome underwent immediate aortic replacement surgery as the definitive intervention. Thirteen (12.6%) patients underwent concomitant aortic valve replacement and ascending aortic replacement. Eleven (84.6%) patients received a biological valve, whereas 2 (15.4%) received a mechanical valve. Eighteen (17.5%) patients underwent a composite aortic root (Bentall) replacement and the aortic valve was resuspended in 14 (13.6%) patients. Hemiarch replacement was performed in 88 (85.4%) cases in addition to ascending aortic replacement, and 7 (6.7%) patients required complete arch replacement.

**Table 1:** Preoperative characteristics for patients with and without malperfusion syndrome

Variables	Malperfusion, *N* = 29 (median, %)	No malperfusion, *N* = 74 (median, %)	*p* -Value
Male	20 (69%)	51 (68.9%)	0.99
Age (y)	58.72 ± 12.1 (41)	59.48 ± 15.16 (53.8)	0.81
Systolic blood pressure (mm Hg)	126.24 ± 32.27 (130)	133.53 ± 35.53 (134)	0.34
BMI (kg/m ^2^ )	29.95 ± 6.74 (30.8)	28.98 ± 5.82 (27.2)	0.47
HCT	39.27 ± 7.85 (40.4)	37.66 ± 6.41 (39)	0.33
Preop creatinine	1.19 ± 0.59 (1.1)	1.24 ± 0.90 (1.1)	0.76
Tamponade	7 (24.1%)	19 (25.7%)	0.87
Prior CAD	4 (13.8%)	10 (13.5%)	0.97
Prior Stroke	2 (6.9%)	3 (4.1%)	0.54
COPD	2 (6.9%)	13 (17.6%)	0.16
Rupture of aorta	2 (6.9%)	5 (6.8%)	0.98
Transferred from outside facility	11 (37.93%)	46 (62.2%)	0.02
Admission–CT interval (min)	1.39 ± 1.33 (0.86)	1.89 ± 2.87 (1.25)	0.36
Admission–incision interval (h)	4.32 ± 2.56 (3.56)	6.37 ± 4.68 (5.50)	0.02

Abbreviations: BMI, body mass index; CAD, coronary artery disease; COPD, chronic obstructive pulmonary disease; CT, computed tomography; HCT, hematocrit.

**Table 2:** Operative management of patients presenting with and without malperfusion syndrome

Variables	Malperfusion group ( *N* = 29) (%)	Nonmalperfused ( *n* = 74) (%)	*p* -Value
Root replacement	2 (6.9)	15 (20.3)	0.10
Bentall procedure	5 (17.2)	13 (17.6)	0.96
Valve-sparing procedure	6 (20.7)	8 (10.8)	0.18
Hemiarch replacement	27 (93.1)	61 (82.4)	0.16
Total arch replacement	1 (3.4)	6 (8.1)	0.39
Descending procedure	0	4 (5.4)	0.20
Concomitant CABG	2 (6.9)	5 (6.8)	0.98
DHCA use	20 (69)	37 (50)	0.08
Antegrade cerebral perfusion	8 (27.6)	22 (29.7)	0.82
Retrograde cerebral perfusion	1 (3.4)	14 (18.9)	0.04
CPB time (min)	184.20 ± 46.35 (180)	192.21 ± 50.54 (186)	0.46
X clamp time (min)	90.51 ± 35.34 (86)	111.70 ± 43.40 (108.5)	0.02

Abbreviations: CABG, coronary artery bypass graft; CPB, cardiopulmonary bypass; DHCA, deep hypothermic circulatory arrest; X clamp, cross clamp.

Unadjusted 30-day mortality for patients presenting with and without malperfusion was 13.8 and 9.5%, respectively. Multivariate regression analysis (adjusting for age, sex, BMI, and race) comparing the malperfusion group to patients with no malperfusion did not reveal a significant difference in perioperative mortality (odds ratio [OR]: 1.53, 95% confidence interval [CI]: 0.40–5.82, *p* = 0.49) ( Table 3 ).

**Table 3:** Postoperative complications in patients with and without malperfusion syndrome

Variables	Malperfusion						
	No ( *n* = 74)		Yes ( *n* = 29)		*p*	Unadjusted OR (95% CI)	Adjusted OR (95% CI) a
	*N*	%	*N*	%			
ICU stay (≥5 d)	36	48.6	15	51.7	0.77	1.13 (0.48–2.67)	1.13 (0.47–2.72)
Vent over 48 h	22	29.7	14	48.2	0.07	2.21 (0.91–5.33)	2.15 (0.83–5.56)
Sepsis	4	5.4	1	3.4	1	0.63 (0.07–5.84)	0.55 (0.05–5.45)
Renal failure requiring dialysis	0	0	2	6.9	0.07		
Reopen for bleeding	10	13.5	5	17.2	0.62	1.33 (0.41–4.30)	1.43 (0.43–4.70)
Postoperative stroke	6	8.1	5	17.2	0.28	2.36 (0.66–8.45)	2.27 (0.60–8.65)
Postoperative HF	6	8.1	2	6.9	1	0.84 (0.16–4.42)	0.74 (0.13–4.11)
3-y mortality	15	20.2	5	17.2	0.72	0.82 (0.27–2.51)	0.78 (0.25–2.44)
30-d mortality	7	9.4	4	13.7	0.49	1.53 (0.41–5.68)	1.53 (0.40–5.82)
Operative mortality	6	8.1	2	6.9	1	0.84 (0.16–4.42)	0.83 (0.15–4.49)
Reoperation	11	14.8	4	13.7	1	0.92 (0.27–3.15)	0.84 (0.23–3.01)
Redo aortic root operation	3	4.0	2	6.9	0.61	1.75 (0.28–11.07)	2.16 (0.30–15.56)

Abbreviations: BMI, body mass index; CI, confidence interval; HF, heart failure; ICU, intensive care unit; OR, odds ratio; Vent, ventilation.

a OR adjusted for age, race, sex, and BMI using multivariate regression.

**Fig. 2:**
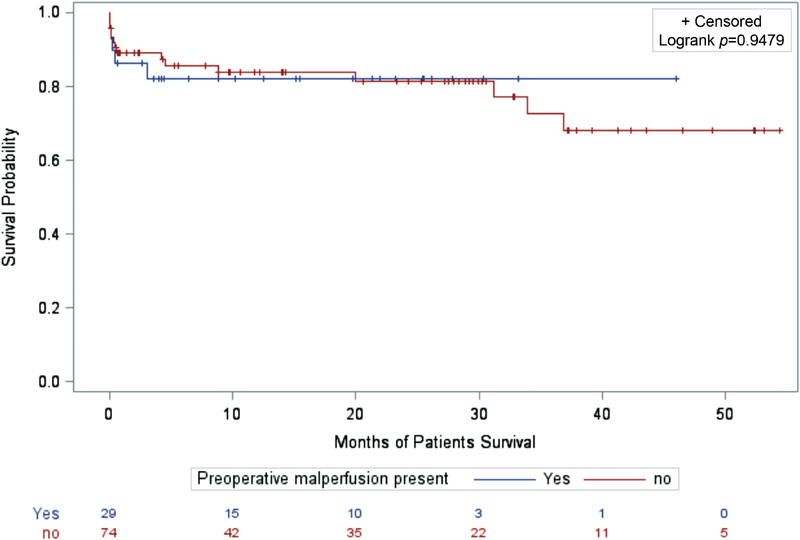
Kaplan–Meier (KM) curve demonstrating long-term survival of patients with and without malperfusion syndrome. No., patients at risk at start of interval; blue, malperfusion; red, nonmalperfusion group.

Causes of 30-day mortality for patients with malperfusion were multiorgan failure (two patients), stroke (one patient), and cardiogenic shock (one patient). Three-year survival for patients with and without malperfusion on Kaplan–Meier analysis was 79.1 and 74.0%, respectively, with no significant difference in long-term mortality ( *p* = 0.94; Fig. 2 ). A proportional hazard analysis did not reveal a significant difference in 3-year survival for patients with and without malperfusion syndrome (hazard ratio: 1.14; 95% CI: 0.40–3.26).

Median postoperative intensive care unit stay for all patients was 4 days and the mean length of hospital stay was 14.3 ± 9 days (median = 9). Five (4.9%) patients required interval redo aortic surgery (2 root, 2 arch, and 1 descending) during long-term follow-up.

## Discussion

There was no difference in our short- to midterm outcomes for patients with or without malperfusion syndrome. When compared with the literature, however, the mortality rate in this cohort is favorable possibly owing to the rapid triage considering that the only significant difference in management for the malperfusion group was a shorter admission-to-incision interval.

Current literature describing large multicenter registries reveals > 15% perioperative mortality for ATAD surgery. 2 8 9 Most studies have shown temporal improvement in surgical mortality for high volume aortic centers, 10 generally as a result of improved surgical technique, surgeon experience, and technical advancement over the years. 10 11 However, early mortality after repair of ATAD complicated by malperfusion remains higher than uncomplicated cases. In a recent, large population-based study comprising 1,159 ATAD patients, Geirsson et al demonstrated using a multivariate regression model that patients with any type of malperfusion were almost four times more likely to die in the early (30-day) postoperative period compared with the general cohort (OR: 3.84, 95% CI: 1.87–7.90, *p* < 0.001). 10 Similarly, in a cohort of 197 patients, a multivariate risk model by Rylski et al demonstrated increased risk of perioperative mortality in patients with ≥ 1 organ malperfusion (OR: 4.74, 95% CI: 1.63–13.80, *p* = 0.004). 12 Another study, reviewing determinants of adverse outcomes in ATAD surgery found patients presenting with malperfusion to have higher risk (OR = 2.95, 95% CI: 1.14–7.67, *p* = 0.026) of an adverse event (stroke or 30-day mortality). 13 Our results demonstrated a nonsignificant difference in early and midterm mortality provided the diagnosis was established rapidly, allowing for rapid triage to the operating room.

Some centers, and this is primarily of historical interest, have described patient-specific staged procedures initially managing malperfusion syndrome (bypass, stenting, and/or fenestration) and performing a delayed aortic replacement once end-organ ischemia is resolved. 3 14 15 Literature describing this algorithm demonstrates equipoise in long-term outcomes of patients who survived the interval delay in surgery and patients who were operated immediately. 14 However, this approach is associated with interval mortality due to rupture and ischemia during the intermission between initial intervention and definitive aortic surgery. On the contrary, none of the patients in our cohort died from frank rupture of the aorta as a consequence of ATAD.

We practice and advocate immediate central aortic surgery for all patients presenting with ATAD regardless of malperfusion syndrome. Our results demonstrate that prompt operation can normalize early and late survival for patients with malperfusion syndrome to those without malperfusion. Patients with malperfusion were rapidly diagnosed and triaged to the operating room in our cohort such that their admission-to-incision interval was significantly shorter than those presenting without malperfusion. This may be explained by the prompt and early realization of ATAD due to explicit signs of malperfusion.

Integration of the ATAD protocol was important in generating a coordinated approach among ED physicians and the cardiac surgery team. The protocol dictated a rapid triage for patients presenting with a high index of suspicion (chest pain, limb numbness/weakness, altered mentation, acute abdominal pain/diarrhea), early and consistent use of POC echocardiogram in the ED, and prioritizing of chest CT scan. A POC echocardiogram performed by the ED physician is helpful in confirming ATAD for patients who have low-moderate suspicion and did not appear to significantly increase the admission-to-incision time interval. Institutional implementation of this protocol was very effective with the majority of cases being triaged and operated within a 5-hour window after presentation to the Yale ED ( Fig. 1 ). The protocol also includes an algorithm to directly transfer patients diagnosed with ATAD from outside medical facilities to the YNHH operating room.

As the majority of patients in our cohort were operated within a 10-hour window ( Fig. 1 ), we do not have a wide range of time points to carry out a robust time-to-intervention based analysis. Further studies with larger sample sizes are required to study the role of time delay in ATAD surgery to validate the effect of time-to-intervention on surgical outcomes of patients suffering from malperfusion.

Our sample size was not adequate to demonstrate statistical significance for difference in early postoperative outcomes. Short-term mortality rates may have achieved significance with a larger sample size; however, we believe that the results highlight potential room for improvement in moving all ATAD patients to the operating room in a shorter time interval as one would for the highest acuity patients.

## Conclusions

Our results albeit limited by a small sample size demonstrated a nonsignificant difference in short-term or midterm mortality rates between the malperfusion group and the nonmalperfused group. Patients presenting with malperfusion underwent a rapid triage to the operating room significantly shortening their “admission–incision” interval. This may have been a major determinant for the comparable outcomes between the two groups; however, further adequately powered studies are required to elaborate and confirm this potential association.
